# Influence of De Novo Malignancies on Long-Term Survival after Lung Transplantation

**DOI:** 10.3390/cancers15154011

**Published:** 2023-08-07

**Authors:** Eloisa Ruiz, Paula Moreno, Francisco Javier Gonzalez, Alba Maria Fernandez, Benito Cantador, Juan Luis Parraga, Angel Salvatierra, Antonio Alvarez

**Affiliations:** Department of Thoracic Surgery and Lung Transplantation, University Hospital Reina Sofia, 14004 Cordoba, Spain; eloisaruiz8@gmail.com (E.R.); pmoreno39@gmail.com (P.M.); fjaviergonzalez@outlook.com (F.J.G.); fernandezgonzalezalbam@gmail.com (A.M.F.); benitocantador@gmail.com (B.C.); jlparragaf@hotmail.com (J.L.P.); asalvati@separ.es (A.S.)

**Keywords:** lung transplantation, malignancies, survival, lung cancer, outcomes

## Abstract

**Simple Summary:**

Lung transplant recipients are at high risk of malignancies. Despite the continuous improvements in lung transplant outcomes over time, limited knowledge exists about the real impact of de novo malignancies developing in lung transplant recipients on their long-term survival. For this reason, we aimed to assess the prevalence of de novo malignancies in a large cohort of lung transplant recipients, their influence on long-term survival, and whether malignancies were an independent risk factor for mortality. We found that 12% of the overall series developed some type of malignancy, with malignancy-related mortality for almost half of the patients developing malignancies. This finding reflects the magnitude of the problem. Furthermore, we observed that de novo lung cancers were the most lethal, affecting the native lungs of recipients receiving single lung transplants. This observation makes it necessary to reconsider performing single lung transplants, especially in patients with chronic obstructive pulmonary disease.

**Abstract:**

(1) Background: Malignancies are an important cause of mortality after solid organ transplantation. The purpose of this study was to analyze the incidence of malignancies in patients receiving lung transplants (LT) and their influence on patients’ survival. (2) Methods: Review of consecutive LT from 1994 to 2021. Patients with and without malignancies were compared by univariable and multivariable analyses. Survival was compared with Kaplan-Meier and Cox regression analysis. (3) Results: There were 731 LT malignancies developed in 91 patients (12.4%) with related mortality of 47% (*n* = 43). Native lung cancer, digestive and hematological malignancies were associated with higher lethality. Malignancies were more frequent in males (81%; *p* = 0.005), transplanted for emphysema (55%; *p* = 0.003), with cyclosporine-based immunosuppression (58%; *p* < 0.001), and receiving single LT (65%; *p* = 0.011). Survival was worse in patients with malignancies (overall) and with native lung cancer. Risk factors for mortality were cyclosporine-based immunosuppression (OR 1.8; 95%CI: 1.3–2.4; *p* < 0.001) and de novo lung cancer (OR 2.6; 95%CI: 1.5–4.4; *p* < 0.001). (4) Conclusions: Malignancies are an important source of morbidity and mortality following lung transplantation that should not be neglected. Patients undergoing single LT for emphysema are especially at higher risk of mortality due to lung cancer in the native lung.

## 1. Introduction

It is well known that solid organ transplant recipients are at increased risk for a variety of cancers [1]. Even though early outcomes after lung transplantation (LT) have improved dramatically over time, malignancies currently represent the second most common cause of death five to ten years after LT. The proportion of patients dying from malignancy increases parallel to survival time, ranging from 3% during the first year post-transplant to 14.5% after five years of survival [2,3,4].

According to the report of the International Society for Heart and Lung Transplantation (ISHLT), up to 17% of lung transplant recipients surviving five years develop malignancies, the second most common cause of late deaths after transplantation [2]. Among recipients of lung transplants, the most common malignancies are non-melanoma skin cancers, lung cancer, and post-transplant lymphoproliferative disease (PTLD) [5].

Lung transplant patients receiving more immunosuppression than other solid organ transplant populations are at increased risk for malignancies in the post-transplant period. Immunosuppressive therapy for lung transplantation is based on a regimen using three drugs: calcineurin inhibitors, antimetabolites, and steroids. Two of these types of agents used for lung transplantation have oncogenic effects. Calcineurin inhibitors, such as cyclosporine and tacrolimus, induce cancer development by increasing levels of the cytokine-transforming growth factor-beta [6,7].

Some infections have also been related to certain malignancies. Most cases of PTLD in lung transplant recipients are associated with EBV infection and high levels of immunosuppression. In those cases of EBV-negative recipients at the time of LT, primary EBV infection after the start of immunosuppressive therapy is related to high risks of PTLD [8].

Therefore, the combination of several risk factors–immunosuppression, age, and exposure to several carcinogens–put the recipients at risk of developing malignancies after LT. However, despite the fact that survival following LT continues to improve, there is limited evidence regarding the potential influence of de novo malignancies on survival [9].

The objective of our study was to analyze the incidence and risk factors of de novo malignancies in patients undergoing LT and whether they have an impact on long-term outcomes and survival after LT.

## 2. Materials and Methods

### 2.1. Study Design

This is an observational analytic retrospective case-control study to determine the rate of malignancies in recipients of a lung transplant and to assess its influence on long-term outcomes and survival. For this purpose, the medical records from the pulmonary transplantation database of 731 consecutive patients transplanted between January 1994 and December 2021 at our Institution were retrospectively reviewed.

### 2.2. Inclusion/Exclusion Criteria

All patients receiving a lung transplant within the study period were initially included. Those patients not surviving for 30 days post-transplant were excluded from the analysis.

### 2.3. Pre-Transplant Assessment

Transplant candidates underwent a thorough assessment including a complete laboratory workup; contrast-enhanced CT scan of the chest, abdomen, and pelvis; complete pulmonary function testing and arterial blood gas analysis; six-minute walk test and cardiopulmonary exercise study; quantitative perfusion lung scan; complete cardiologic assessment including electrocardiogram, echocardiogram, right heart catheterization in patients above age 45, and additional left heart catheterization for coronary angiography in cases with risk factors for coronary artery disease; immunotyping; and complete microbiological and serological status.

Patients were selected for lung transplantation after a multidisciplinary evaluation according to International Guidelines [10].

### 2.4. Donor Selection and Surgical Procedure

Donors met the standard acceptability criteria in all cases [11]. The organ procurement was performed following the standard technique of combined cardiopulmonary extraction. In the recipients, a standard surgical procedure was followed to implant the lung grafts [12].

### 2.5. Recipient Post-Transplant Assessment and Management

Recipients were given a daily follow-up until discharge, then weekly in the first month, every other week over the next five months, monthly until the first 12 months post-transplant and every three months thereafter. A scheduled bronchoscopic assessment of the airway was performed immediately after transplantation, before weaning, and before hospital discharge. Surveillance bronchoscopies with bronchoalveolar lavage and transbronchial biopsies were performed at one, three, six, and twelve months post-transplant. Additional bronchoscopies were performed whenever a clinical suspicion of infection or rejection arose. Annually, all patients underwent a non-contrast chest CT. In the event of a nodule or lung mass being identified, a tailored approach to diagnosis was followed. If malignancy was diagnosed, a multidisciplinary assessment was discussed with the Institutional Board of Thoracic Tumours, which comprises pulmonologists, thoracic surgeons, medical and radiation oncologists, radiologists, and others.

### 2.6. Immunosuppression

Immunosuppression included a calcineurin inhibitor, either tacrolimus [Prograf^®^; Fujisawa. Killorglin Co., Kerry, Ireland] or cyclosporine [Sandimmun^®^; Novartis, Basle, Switzerland]; an antiproliferative agent, either mycophenolate [Cellcept^®^; Roche Lab. Inc., Nutley, NJ, USA] or azathioprine [Imurel^®^; Medeva Pharma, Madrid, Spain]; and corticosteroids [Dezacor^®^; Hoechst Marion Roussel, Barcelona, Spain]. Mycophenolate was discontinued if a malignancy was diagnosed, and the tacrolimus dose was lowered.

Induction therapy was used in some patients [Basiliximab^®^, Novartis, Basle, Switzerland]. 

### 2.7. Management of Infections

Antimicrobial therapy was given based on antibiotic sensitivities from preoperative sputum cultures of the recipient and from the donor broncho-aspirate. Viral and fungal prophylaxis was established following standardized protocols [13]. Airway fungal colonizations were treated with systemic voriconazole for two weeks and aerosolized amphotericin B for three months. In addition, oral nystatin was administered to cystic fibrosis recipients.

### 2.8. Data Collection

Donor data included age, gender, and smoking habits. Recipient preoperative data included age, gender, smoking habits, indication for lung transplantation, donor/recipient CMV status, donor/recipient EBV status, and comorbidities. Surgical and early postoperative data included type and side of lung transplant, number of acute rejection episodes, type of immunosuppression, and hospital stay. Late postoperative data included the number and site of malignancies, tumor stage, length of time between LT and diagnosis of malignancy and death, overall mortality, and survival. Data were analyzed and compared between recipients with and without malignancies by univariable and multivariable analyses.

### 2.9. Statistical Analysis

#### 2.9.1. Univariable Analysis

We compared recipients with vs. without malignancies by either Pearson’s χ^2^ or Fisher’s exact test for categorical variables and either unpaired *t*-test or Mann-Whitney U-test for quantitative variables. Survival was analyzed and compared using the Kaplan–Meier method and log-rank test.

#### 2.9.2. Multivariable Analysis

To identify independent predictors of mortality, those variables with *p* values below 0.1 in the univariable tests entered into a multivariable Cox regression analysis, and *p* values below 0.05 in the final model were judged to be independent predictors of mortality.

Continuous variables are expressed as means ± standard deviation. Categorical variables are expressed as counts and proportions with 95% confidence intervals (95%CI). Differences with *p* values < 0.05 were considered significant. The statistical analysis was performed using SPSS (SPSS 20.0 for Mac: SPSS, Inc., Chicago, IL, USA).

## 3. Results

There were 731 patients: 509 (70%) males and 222 (30%) females, with a mean age of 47 ± 16 years old (4–68 years). Transplant indications were chronic obstructive pulmonary disease (COPD) in 276 patients (38%), pulmonary fibrosis in 167 (23%), cystic fibrosis in 150 (20%), bronchiectasis in 23 (3%), re-transplants in 10 (1%), and other indications in 105 patients (15%).

Ninety-one patients developed some malignancy following the lung transplant (12.4%). Forty-three cases died from their neoplasm, comprising almost 6% of overall cases, with a malignancy-related mortality of 5.8% of the overall group and a 47% rate of all neoplasms.

When exploring differences between patients with and without malignancies, we observed that patients with malignancies were older and more frequently males (81%). Additionally, more than half of malignancies appeared in patients transplanted for emphysema, with cyclosporine as primary immunosuppression, and those receiving single lung transplants (Table 1).

**Figure 1:**
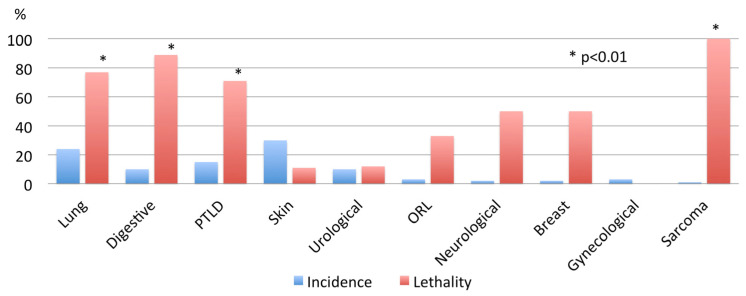
Overall incidence and lethality of malignancies arising after lung transplantation. Lung cancer: 22(24%); lethality 17/22 (77%). Digestive neoplasms: 9 (10%); lethality 8/9 (89%). Hematological (post-transplant lymphoproliferative disease—PTLD): 14 (15%); lethality 14/14 (71%). Skin neoplasms (non-melanoma): 27 (30%); lethality 3/27 (11%). Urological: 8 (9%); lethality 1/18 (12%). ORL: 3 (3%); lethality 1/3 (33%). Neurological (brain): 2 (2%); lethality 1/2 (50%). Breast cancer: 2 (2%); lethality 1/2 (50%). Gynecological malignancies: 3 (3%); lethality 0/3 (0%). Sarcomas: 1 (1%); lethality 1/1 (100%).

Figure 1 depicts the incidence and lethality of all malignancies in our series: skin cancers are the most frequent, but their lethality is very low. On the contrary, lung cancer appeared in up to 20% of recipients, being associated with a high lethality rate. Also, digestive, post-transplant lymphoproliferative disease (PTLD), and sarcomas were associated with high lethality rates.

**Figure 2:**
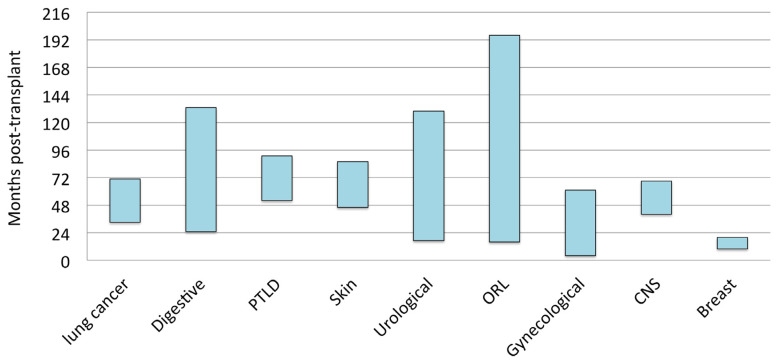
Post-transplant period until diagnosis of malignancy. Lung cancer: 52 ± 42 months [95%CI: 33–71]. Digestive neoplasms: 79 ± 70 months [95%CI: 25–133]. Hematological (post-transplant lymphoproliferative disease—PTLD): 72 ± 34 months [95%CI: 52–91]. Skin neoplasms (non-melanoma): 66 ± 51 months [95%CI: 46–86]. Urological: 74 ± 67 months [95%CI: 17–130]. ORL: 89 ± 43 months [95%CI: 16–196]. Gynecological malignancies: 33 ± 11 months [95%CI: 4–61]. Central nervous system (CNS): 54 ± 36 months. Breast cancer: 13 ± 10 months.

The post-transplant period time until the diagnosis of malignancy varied, from a wide period for urological and ORL neoplasms to a shorter period for lung cancer, PTLD, and melanomas. Focusing on lung and digestive cancers, our experience showed they appeared after two years following the transplant (Figure 2).

**Figure 3:**
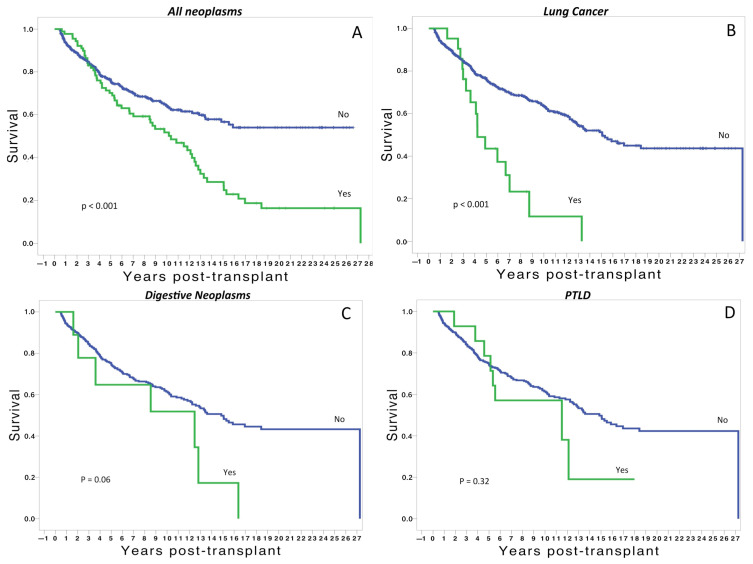
(**A**) Overall post-transplant survival of patients with and without malignancies. (**B**) Survival of patients developing post-transplant lung cancer vs. those without post-transplant lung cancer. (**C**) Survival of patients developing post-transplant digestive neoplasms vs. those without digestive neoplasms. (**D**) Survival of patients developing post-transplant lymphoproliferative disease vs. those without post-transplant lymphoproliferative disease.

In relation to the survival analysis, we observed that patients developing malignancies have significantly worse survival than those without malignancies (Figure 3A). This is especially true for recipients developing lung cancer after the transplant, with a significant decline in the survival curve within the first five years post-transplant (Figure 3B). Regarding digestive neoplasms, survival was worse than those without neoplasms, but the analysis did not reach significant differences (Figure 3C). Similarly, there was a trend to worse survival in transplants with hematological malignancies, but without significant differences (Figure 3D).

To elucidate the real impact of malignancies on survival, we developed a Cox model including other factors related to survival, such as type of transplant, indication, age of donors and recipients, immunosuppression, recipients’ gender, and donor smoking habits. In the final model, those recipients with cyclosporine-based immunosuppression had an almost two-fold risk for mortality, and those developing de novo lung cancer had a 2.6-fold higher mortality risk than those without lung cancer (Table 2).

## 4. Discussion

In the present study, we observed that de novo malignancies occur in 12.4% of patients undergoing lung transplantation, with malignancy-related mortality of 5.8% and a lethality rate of 47%. These figures demonstrate that the development of malignancies following lung transplantation is a devastating complication influencing long-term survival. This is especially true for lung cancer, digestive neoplasms, and PTLD. Age, male gender, patients transplanted for COPD, and single-lung transplantation appear to be associated with the development of de novo malignancies. Variables independently associated with reduced survival were cyclosporin-based immunosuppression and de novo lung cancer.

We observed that lung transplant recipients developing malignancies were older and, more frequently, males (81%). It is well known that, in the general population, the risk for cancer increases with age. After the sixth decade, the risk for lung cancer doubles from 1% to 2.3% in men and 0.8% to 1.7% in women [14]. As demonstrated in the present series, this association also applies to lung transplant recipients.

In the present series, skin cancers were the most frequent malignancies, but their lethality was very low. On the contrary, lung cancer appeared in up to 20% of recipients, associated with a high lethality rate. Also, digestive, post-transplant lymphoproliferative disease (PTLD), and sarcomas were associated with high lethality rates.

Malignancies represent the third cause of death after the first year post-transplant [2]. From the date of lung transplantation, the proportion of patients dying from malignancy increases with time, ranging from 3% at one year post-transplant to 14% after five years [2]. In our series, we observed that patients developing malignancies have significantly worse survival than those without malignancies (Figure 3A). This is especially true for recipients developing lung cancer after the transplant, with a significant decline in the survival curve within the first five years post-transplant (Figure 3B). Similarly, there was a trend to worse survival in recipients developing digestive and hematological neoplasms, but without significant differences (Figure 3C,D).

It is important to consider the time of development of neoplasms following LT. In our experience, the post-transplant period time until the diagnosis of malignancy varied, from a wide period for urological and ORL neoplasms to a shorter period for lung cancer, PTLD, and skin neoplasms. Focusing on lung and digestive cancers, we found they appeared two years after the transplant (Figure 3). This finding is remarkable and highlights the importance of developing strategies to identify at-risk patients to promote appropriate screening tools. Regarding lung cancer, we suggest a close follow-up with chest CT scans from the first year post-transplant, especially in patients with emphysema or pulmonary fibrosis receiving a single lung transplant [15].

Skin cancers are the most common malignancies after lung transplantation [1,16], accounting for up to 50% of all cancers reported in the post-transplant population. Squamous cell carcinoma (SCC) is the most common, with a 100–200-fold increased risk compared to the general population [16]. Non-melanoma skin cancers appear more frequently in younger recipients than in the non-transplant population and behave more aggressively, with higher rates of recurrence and mortality [17]. Similarly, in our series, skin cancer was the most frequent neoplasm, but its lethality rate was extremely low, probably due to the fact that the majority of cases were SCC and appeared in older recipients. The association of voriconazole with the development of skin cancers has been reported [18]. Unfortunately, from the data analyzed in the present series, we were unable to identify such an association.

The incidence of PTLD after lung transplantation has been reported to be 3–9% and is associated with worse long-term survival [19]. The majority of cases are associated with EBV infection, likely related to the donor lymphoid tissue in the allograft containing latent EBV and the increased intensity of the post-transplant immunosuppression regimen [8]. Similarly, in our experience, PTLD developed more frequently in those transplants when the donor and/or the recipient were EBV-positive. PTLD can develop at any time point after transplantation, but early cases are more frequently seen in younger recipients and in those recipients who are EBV-negative, acquiring the infection from the donor. The late onset of PTLD is generally associated with a worse prognosis [19,20]. Immunosuppression reduction alone has a response rate of up to 45%, but this strategy is associated with a significant risk of rejection and graft loss [21]. In fact, it has been reported that chronic lung allograft dysfunction, rather than PTLD, is the leading cause of death in lung transplant patients with PTLD.

Lung cancer is more commonly seen in lung transplant recipients than in the general population [1,5], with LT recipients having up to five-fold increased risk of lung cancer compared to the general population, with a reported incidence of 1–9% [22,23]. Recently, we reported a 7% rate of lung cancer arising in COPD patients receiving single lung transplants, with a significant decline of long-term survival. In that series, five lung cancer patients were in stages I/II and underwent surgical resection, whereas the remaining six cases underwent chemo/radiotherapy [24,25].

In our experience, most cases of lung cancer in lung transplant patients arise in the native lung of single lung transplant recipients, with rates ranging from 1% to 9% [5,26,27]. In the present series, the development of lung cancer arose in 22 recipients (24% of all neoplasms). Among them, 17 patients died due to lung neoplasm (lethality rate of 77%). In contrast, lung cancer in the allograft after single or bilateral lung transplantation is unusual [5]. In our series, none of the lung cancers appeared in the allograft. It is clear that single lung recipients appear to be at the highest risk for lung cancer, as this procedure leaves behind a native lung at risk [1,5,22,24,26,28].

It is well known that, in the general population, early-stage lung cancer has a better prognosis than advanced stages. However, this prognosis does not apply to recipients of lung transplants. In fact, a recent report from the US Scientific Registry for Transplant (SRTR) observed that the effects of treatment in lung transplant recipients are generally poorer than lung cancer treated in the general population, even though diagnosed at earlier stages [22]. Our present experience confirms these observations, and they are probably due to the deleterious effects of immunosuppression on promoting aggressive tumor behavior and metastasis. Unfortunately, as we did not assess the incidence of lung cancer in our general population, we could not make comparisons between lung transplant and non-lung transplant patients.

The treatment strategies include a reduction of immunosuppression with the inclusion of an mTOR inhibitor, given their antineoplastic effect [29]. Unfortunately, the majority of lung transplant recipients have poor general conditions and limited pulmonary reserve to be considered candidates for lung resection or conventional chemotherapy and/or radiation [30]. For these reasons, the careful surveillance of any change in the native lung after SLT is of paramount importance for the early detection of lung cancer. 

The present study has limitations inherent to retrospective analyses and represents the experience of a single institution. Some biases should be considered, given the long period of study, with changes in the transplant procedure and perioperative management over time. Missing data and interactions with variables not studied, such as infections or CLAD, could have had an influence on our results. In addition, the prevalence of neoplasms in our Spanish transplant cohort might differ from that of other populations. Finally, as we did not collect data from the general population, comparisons among transplant and non-transplant individuals were not performed.

## 5. Conclusions

In conclusion, de novo malignancies are an important source of morbidity and mortality following lung transplantation that should not be neglected. Especially those single lung transplants for COPD are at higher risk of mortality due to lung cancer in the native lung.

## Figures and Tables

**Table 1:** Differences between transplant patients with or without malignancies.

		Malignancies NO (*n* = 640) YES (*n* =91)				*p*
			95%CI		95%CI	
Recipient age (years)		46 ± 16		51 ± 13		0.01
Donor age (years)		42 ± 17		38 ±16		0.03
Recipient gender	n (%)					
Male		434 (68)	64–72	74 (81)	73–89	
Female		205 (32)	28–36	17 (19)	11–27	0.005
Donor gender	n (%)					
Male		291 (49)	44–53	60 (66)	56–76	
Female		293 (50)	46–54	31 (34)	24–44	0.001
Extended donor	n (%)	212 (33)	30–39	24 (26)	17–35	0.06
Indication for LT	n (%)					
Emphysema		226 (35)	31–39	50 (55)	45–65	
Cystic Fibrosis		138 (21)	18–24	11 (12)	6–18	
Pulmonary Fibrosis		144 (22)	19–25	23 (25)	16–34	
Bronchiectasis		21 (4)	3–5	2 (2)	0–4	
Other		110 (18)	15–21	5 (6)	1–11	0.003
Donor smoking	n (%)	108 (17)	14–20	9 (10)	4–16	0.62
EBV D/R	n (%)					
−/−		15 (3)	2–4	7 (8)	3–13	
−/+		62 (10)	8–12	3 (3)	0–6	
+/−		54 (8)	6–10	3 (3)	0–6	
+/+		173 (27)	24–30	32 (35)	25–45	
?/−		44 (7)	5–9	9 (10)	4–16	
?/+		292 (45)	41–49	37 (41)	31–51	0.03
Initial IS	n (%)					
CS + AZA + Steroids		113 (17)	14–20	31 (34)	24–44	
FK+ AZA+ Steroids		32 (5)	4–6	0		
CS + MMF + Steroids		123 (19)	16–22	23 (25)	16–34	
FK + MMF + Steroids		372 (58)	54–62	37 (41)	31–51	<0.001
Calcineurin inhib. based IS	n (%)					
CS		236 (37)	33–41	54 (59)	49–69	
FK		404 (63)	59–67	37 (41)	31–51	<0.001
Changes in IS	n (%)					
Yes/No		168/472		68/23		<0.001
FK to CS		117 (18)	15–21	38 (42)	32–52	0.07
MMF to AZA		51 (8)	6–10	30 (33)	23–43	0.01
CMV infection/disease n (%)						
<1 month post-LT		21 (3)	2–4	11 (12)	6–18	0.07
2–3 months post-LT		29 (5)	4–6	16 (17)	9–25	0.09
>3 months post-LT		48 (8)	6–10	23 (25)	16–34	0.06
Total AR episodes (n)		1.0 ± 1.14	1.1–1.3	1.1 ± 1.05	0.9–1.3	0.55
AB0 group	n (%)					
0	276 (38)	239 (37)	33–41	37 (40)	30–50	
A	354 (48)	310 (49)	45–53	44 (48)	38–58	
B	75 (10)	67 (10)	8–12	8 (9)	3–15	
AB	25 (3)	24 (4)	3–5	2 (3)	0–6	0.02
Type of LT	n (%)		44–52 47–55		55–75 25–45	
Single LT		308 (48)		59 (65)		
Double LT		327 (51)		32 (35)		
Combined liver + Double LT		5 (0.7)		0		0.01

AR: acute rejection episodes; AZA: azathioprine; CMV: cytomegalovirus; CS: cyclosporine; D/R: donor/recipient; EBV: virus Epstein-Barr; FK: tacrolimus; IS: immunosuppression; LT: lung transplantation; MMF: mycophenolate mofetil.

**Table 2:** Independent predictors of mortality in the present series (Cox regression analysis).

	OR	95%CI	*p*
Cyclosporine-based immunosuppression	1.8	1.3–2.4	<0.001
De novo lung cancer	2.6	1.5–4.4	<0.001

## Data Availability

Due to patients’ privacy and ethical restrictions, an anonymized dataset can be shared on reasonable request to the corresponding author.
